# Multiple polymerase gene mutations for human adaptation occurring in Asian H5N1 influenza virus clinical isolates

**DOI:** 10.1038/s41598-018-31397-3

**Published:** 2018-08-30

**Authors:** Yasuha Arai, Norihito Kawashita, Kozue Hotta, Phuong Vu Mai Hoang, Hang Le Khanh Nguyen, Thach Co Nguyen, Cuong Duc Vuong, Thanh Thi Le, Mai Thi Quynh Le, Kosuke Soda, Madiha S. Ibrahim, Tomo Daidoji, Tatsuya Takagi, Tatsuo Shioda, Takaaki Nakaya, Toshihiro Ito, Futoshi Hasebe, Yohei Watanabe

**Affiliations:** 1 Department of Infectious Diseases, Graduate School of Medical Sciences, Kyoto Prefectural University of Medicine, Kyoto, Japan; 2 Department of Viral Infections, Research Institute for Microbial Diseases, Osaka University, Osaka, Japan; 3 Graduate School of Science and Engineering, Kindai University, Osaka, Japan; 4 Graduate School of Pharmaceutical Science, Osaka University, Osaka, Japan; 5 Vietnam Research Station, Center for Infectious Disease Research in Asia and Africa, Institute of Tropical Medicine, Nagasaki University, Hanoi, Vietnam; 6 Laboratory of Veterinary Public Health, Department of Veterinary Medical Sciences, Graduate School of Agricultural and Life Sciences, The University of Tokyo, Tokyo, Japan; 7 Department of Virology, National Institute of Hygiene and Epidemiology, Hanoi, Vietnam; 8 Avian Zoonosis Research Center, Faculty of Agriculture, Tottori University, Tottori, Japan; 9 Department of Microbiology and Immunology, Faculty of Veterinary Medicine, Damanhour University, Damanhour, Egypt

## Abstract

The role of the influenza virus polymerase complex in host range restriction has been well-studied and several host range determinants, such as the polymerase PB2-E627K and PB2-D701N mutations, have been identified. However, there may be additional, currently unknown, human adaptation polymerase mutations. Here, we used a database search of influenza virus H5N1 clade 1.1, clade 2.3.2.1 and clade 2.3.4 strains isolated from 2008–2012 in Southern China, Vietnam and Cambodia to identify polymerase adaptation mutations that had been selected in infected patients. Several of these mutations acted either alone or together to increase viral polymerase activity in human airway cells to levels similar to the PB2-D701N and PB2-E627K single mutations and to increase progeny virus yields in infected mouse lungs to levels similar to the PB2-D701N single mutation. In particular, specific mutations acted synergistically with the PB2-D701N mutation and showed synergistic effects on viral replication both in human airway cells and mice compared with the corresponding single mutations. Thus, H5N1 viruses in infected patients were able to acquire multiple polymerase mutations that acted cooperatively for human adaptation. Our findings give new insight into the human adaptation of AI viruses and help in avian influenza virus risk assessment.

## Introduction

The influenza A virus genome consists of eight single-stranded, negative-sense, RNA segments. In the virion, each viral RNA (vRNA) segment is coated by viral nucleoprotein (NP) and has a viral polymerase complex bound at its 5′ and 3′ termini, to form a ribonucleoprotein (vRNP) for viral replication. The viral polymerase complex consists of three subunits, encoded by the viral PB2, PB1 and PA genes. The polymerase complex is one of the main determinants affecting the host range of influenza viruses^1–4^, and adaptation mutations in the polymerase complex are vital for efficient viral replication in a new host^2,5^. Several adaptation mutations have been identified in the PB2 gene in seasonal human influenza viruses and in AI viruses that have caused occasional human infections. The best known human adaptation polymerase mutation is the PB2-E627K substitution, which has been identified in several human H5N1 and H7N9 isolates and in one human H7N7 isolate^6–9^. The PB2-E627K mutation is correlated with increased H5N1 replication and virulence in mammals and also allows the virus to replicate efficiently in the human upper respiratory tract, where the temperature is slightly lower than in the lower respiratory tract^7,10^. Other mutations in PB2, PB1 and PA also influence the host range of influenza viruses; e.g., PB2-D701N^11,12^, PB2-K526R^8^, PB2-Q591K^13^, PB2-E192K and -K702R^14^, PB2-I147T/K399T/A588T^15^, PB2-E158G^16^, PB1-L13P/S678N^11^, PB1-N105S^14^, PB1-D175N/K198R^17^ and PA-K356R^18^. The mechanism of mammalian adaptation due to these mutations remains largely unknown, although some of the mutations reportedly modified interaction between polymerase and host factors, contributing to host adaptation^19,20^.

Since its emergence in China in 1996, highly pathogenic H5N1 AI virus has caused 860 confirmed human infections with 454 deaths (as of 27 September 2017, according to WHO; http://www.who.int/). H5N1 viruses have become endemic in birds in some countries; e.g., China, Vietnam, Cambodia, Indonesia and Egypt. Continuous circulation has allowed H5N1 viruses to diverge genetically to form phylogenetically distinct clades (designated clades 0 to 9) in different geographic areas^4^. H5N1 viruses have moved across the borders of Southern China, Vietnam and Laos, with different H5N1 clades (i.e., clades 1.0, 1.1, 1.1.1, 1.1.2, 2.3.2, 2.3.2.1, 2.3.4, 2.3.4.1, 2.3.4.2, 2.3.4.3, 7.0, 7.1 and 7.2) circulating in the field^21–23^ and associated with a high number of H5N1 human infections. This geographic area is regarded as a hot-spot for H5N1 virus evolution.

Although some H5N1 human isolates contain known PB2 adaptation mutations, most are PB2-E627K and some are PB2-D701N as described above. However, other H5N1 strains isolated from human infections in Asia do not carry previously identified adaptation mutations. This indicated that there are currently unknown adaptation mutations in the H5N1 polymerase complex that enable viral replication in human cells. Therefore, identification of naturally occurring, but currently unknown, polymerase adaptation mutations for influenza virus replication in infected patients should provide valuable data for H5N1 pandemic risk assessment.

Our bioinformatics study previously identified several human adaptation mutations in H5N1 clade 2.2.1 viruses that were unique to Egypt^17,24^. In this study, we extended that study to other H5N1 clades and carried out a database search of H5N1 clade 1.1, clade 2.3.2.1, and clade 2.3.4 viruses that had circulated in geographically close areas of Southern China, Vietnam and Cambodia during 2008–2012. We identified here novel polymerase mutations in clade 1.1, clade 2.3.2.1 and clade 2.3.4 virus strains isolated from humans in these areas. These mutations acted alone and/or together in the genetic background of both Asian and Middle Eastern H5N1 clades to optimize human infection. These results give new insight into the adaptation of AI viruses for human infection and should help in virus surveillance efforts.

## Results

### Analysis of human adaptation mutations in the polymerase complex of Asian H5N1 Nviruses

Bioinformatics allows us to identify novel mutations in avian H5N1 viruses that enable efficient viral replication in mammals. However, identification of putative adaptation mutations that differ from the consensus sequence is challenging when viruses in diverse H5N1 genetic lineages are analyzed, since rare mutations may be masked. In fact, there has been movement and (co) circulation of distinct H5N1 clades in the field in East and Southeast Asia^4,21–23,25,26^.

To avoid such potential problems, we conducted a database search targeting a geographically and chronologically close group of PB2, PB1, and PA polymerase and NP nucleoprotein gene sequences in human and avian H5N1 strains isolated in Southern China, Vietnam and Cambodia during 2008–2012. The nucleotide sequences were downloaded from the National Center for Biotechnology Information (NCBI) Influenza Virus Resource (www.ncbi.nlm.nih.gov/genomes/FLU/). This yielded a total of 24 clade 1.1, clade 2.3.2.1 and clade 2.3.4 human virus PB2, PB1, PA and NP sequences, which included all the public database available sequences from human viruses in Asia during this period (as of October, 2017) and a total of 198 avian virus PB2, PB1, PA and NP sequences in the corresponding clades. Although a few sequences from human influenza viruses in these clades were isolated later and were available in the GISAID EpiFlu database (https://www.gisaid.org/) at the time of our database search, only sequences isolated in 2008–2012 were used in this study.

The consensus sequences of the polymerase and NP genes were determined by aligning all the sequence data of the vRNA segments carrying those genes. We then searched the sequence data set for polymerase and NP genes with amino acid mutations that had been presumably selected in H5N1-infected patients. Mutations in the polymerase and NP gene sequences of human and avian viruses were identified by comparing each gene sequence to its consensus sequence. Based on their prevalence in human and avian viruses, we identified a total of 33 single mutations (9 in PB2, 8 in PB1, 14 in PA and 2 in NP) that were either only in human viruses or were more prevalent in human viruses than in avian viruses as putative human adaptation mutations in clade 1.1, clade 2.3.2.1, and clade 2.3.4 viruses (Table 1). Some mutations were the only mutation in a vRNA segment in some strains, some mutations were only found together with one or more other mutations in a vRNA segment in some strains, and some mutations were the only mutation in a vRNA segment in some strains and with one or more other mutations in that vRNA segment in other strains (Table S1). Therefore, we searched for multiple mutations in viruses isolated from patients and identified 13 viruses with multiple mutations. In all, 49 single and multiple mutations were investigated in this study.

**Table 1:** Prevalence of H5N1 polymerase mutations in human and avian viruses identified in the database search in this study.

Segment (no. of strains with mutation)	Host of virus with mutation	Mutation	% of strains with mutation (no. of strains)^a^	
			Human viruses	Bird viruses
PB2 (9)	Only in human viruses	H134R	12.5 (3)	0 (0)
		K190R	8.3 (2)	0 (0)
		M315I	8.3 (2)	0 (0)
		D701N	8.3 (2)	0 (0)
	More prevalent in human viruses than in avian viruses	T81A	12.5 (3)	1.5 (3)
		V344M	16.7 (4)	3.0 (6)
		L618M	16.7 (4)	0.5 (1)
		Y658H	12.5 (3)	2.0 (4)
		G685R	8.3 (2)	0.5 (1)
PB1 (8)	Only in human viruses	T156V	8.3 (2)	0 (0)
	More prevalent in human viruses than in avian viruses	T21S	8.3 (2)	0.5 (1)
		M111I	12.5 (3)	0.5 (1)
		N314S	8.3 (2)	2.0 (4)
		N328K	8.3 (2)	0.5 (1)
		D398E	8.3 (2)	0.5 (1)
		M744T	8.3 (2)	0.5 (1)
		K745R	8.3 (2)	0.5 (1)
PA (14)	Only in human viruses	M86V	8.3 (2)	0 (0)
		F562Y	8.3 (2)	0 (0)
	More prevalent in human viruses than in avian viruses	S65P	8.3 (2)	2.0 (4)
		T85A	8.3 (2)	0.5 (1)
		N115S	8.3 (2)	0.5 (1)
		N222S	8.3 (2)	0.5 (1)
		A224P	8.3 (2)	2.0 (4)
		L226F	8.3 (2)	0.5 (1)
		Y305F	8.3 (2)	0.5 (1)
		A343T	16.7 (4)	0.5 (1)
		D394G	12.5 (3)	0.5 (1)
		E610D	8.3 (2)	2.0 (4)
		E613V	16.7 (4)	0.5 (1)
		A689S	8.3 (2)	0.5 (1)
NP (2)	More prevalent in human viruses than in avian viruses	T430A	12.5 (3)	2.5 (5)
		L466F	8.3 (2)	1.5 (3)

^a^The sequences of 24 human virus strains and 198 avian virus strains isolated in Southern China, Vietnam and Cambodia during 2008–2012 and in the NCBI Influenza Virus Resource were used in this study.

### Effect of H5N1 polymerase mutations on viral polymerase activity

To investigate the effect(s) of the mutations on viral polymerase activity, we carried out minigenome assays of polymerases with these mutations in the genetic background of A/Vietnam/HN31432/2008 (VN/HN). VN/HN is a representative H5N1 clade 2.3.4 strain^27^ with no known human adaptation mutations (e.g., PB2-E627K and PB2-D701N) in its HA and polymerase genes. VN/HN was used as the genetic background in this study because most of the identified mutations (13/24) were in clade 2.3.4, clade 2.3.4.1 or clade 2.3.4.3 viruses (Table S1). Human 293T cells and avian QT-6 cells were used for this study. For 293T cells, polymerase activity at both 33 and 37 °C (the temperature of the human upper and lower respiratory tract, respectively) was assayed. For QT-6 cells, polymerase activity was assayed at 37 °C to allow the results to be compared with those for 293T cells at the same temperature. Among the identified mutations, the PB2-D701N single mutation has been reported to increase the replication of avian H5N1 viruses in mammals^12,28,29^ and, therefore, was included in this study for comparison with other single and multiple mutations.

**Figure 1:**
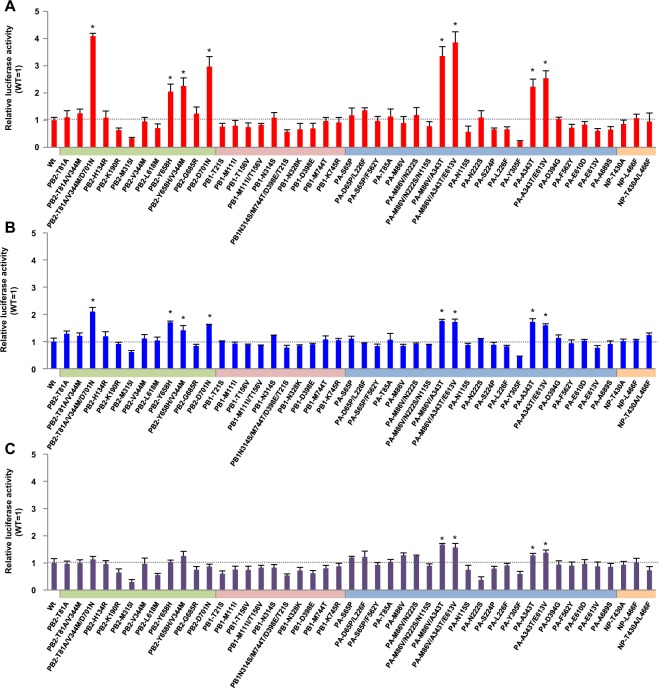
Effect of mutations on H5N1 clade 2.3.4 virus polymerase activity. Human 293T cells and avian DF-1 cells were transfected with plasmids expressing VN/HN PB2, PB1, PA or NP with the indicated single or multiple mutations, a human or chicken polymerase I-driven plasmid expressing a vRNA-oriented luciferase reporter gene, and a plasmid expressing *Renilla* luciferase as an internal control. After 48 h incubation at 33 or 37 °C, luciferase activities were measured, normalized to the internal *Renilla* luciferase activity, and expressed relative to the results for VN/HN (wt). (**A**) VN/HN polymerase activity at 37 °C in 293T cells. (**B**) VN/HN polymerase activity at 33 °C in 293T cells. (**C**) VN/HN polymerase activity at 37 °C in DF-1 cells. Colors on each x-axis indicate the different virus genes. An asterisk indicates a *P* value < 0.01 (ANOVA with Tukey’s multiple comparison test). Asterisks for mutations with negative effects on polymerase activity were omitted for clarity.

Minigenome assays showed that several single and multiple mutations in PB2 and PA increased H5N1 VN/HN polymerase activity in human 293T cells; e.g., PB2-T81A/V344M/D701N, PB2-Y658H, PB2-Y658H/V344M, PB2-D701N, PA-M86V/A343T, PA-M86V/A343T/E613V, PA-A343T and PA-A343T/E613V significantly increased polymerase activity at 37 °C (Fig. 1A), although a few other mutations reduced the polymerase activity. Several multiple mutations acted synergistically to produce a greater increase in polymerase activity than the single mutations. The PB2-T81A/V344M/D701N triple mutation produced up to 4.1-fold higher polymerase activity than wild-type PB2 and significantly higher polymerase activity than the known PB2-D701N human adaptation mutation (Fig. S1A, P < 0.01 by ANOVA with Tukey’s multiple comparison test). The PA-M86V/A343T double mutation also had a synergistic effect, with a 3.9-fold higher polymerase activity than wild-type PA and significantly higher polymerase activity than PA-A343T, which also increased polymerase activity (Fig. S1A, P < 0.01 by ANOVA with Tukey’s multiple comparison test). In each case, the effect of the multiple mutation in increasing polymerase activity was greater than the increase produced by the PB2-D701N single mutation. Similar results were obtained at 33 °C, although the effects were more moderate (Figs 1B and S1B). The mutations in PB2 increased polymerase activity in a human cell-specific manner: the effects were significantly less in avian cells (Figs 1C and S1C). In addition, the mutations increased polymerase activity at both 37 and 33 °C specifically in human cells in the genetic background of A/duck/Egypt/D1Br/2007, which is a representative clade 2.2.1 strain that is geographically unique to Egypt^24,30^ (Fig. S2A‒C). Taken together, these results suggested that the mutations selected by viral growth in patients acted, separately and together, to increase H5N1 polymerase activity in human cells.

### Effect of H5N1 polymerase mutations on viral replication in human airway epithelial cells

We next investigated the effect(s) of the polymerase mutations on the replication kinetics of clade 2.3.4 viruses in human cells using recombinant viruses generated by reverse genetics in the VN/HN genetic background. Based on their polymerase activity in human cells, we selected seven of the mutations that produced significant increases in polymerase activity (i.e., PB2-T81A/V344M/D701N, PB2-Y658H, PB2-Y658H/V344M, PB2-D701N, PA-A343T, PA-M86V/A343T and PA-M86V/A343T/E613V) for further study and rescued replicating viruses carrying these mutations. In addition, viruses carrying either a putative negative control mutation (PB2-G685R) that had little effect on polymerase activity or a representative human adaptation mutation (PB2-E627K) were included in this study for comparison.

**Figure 2:**
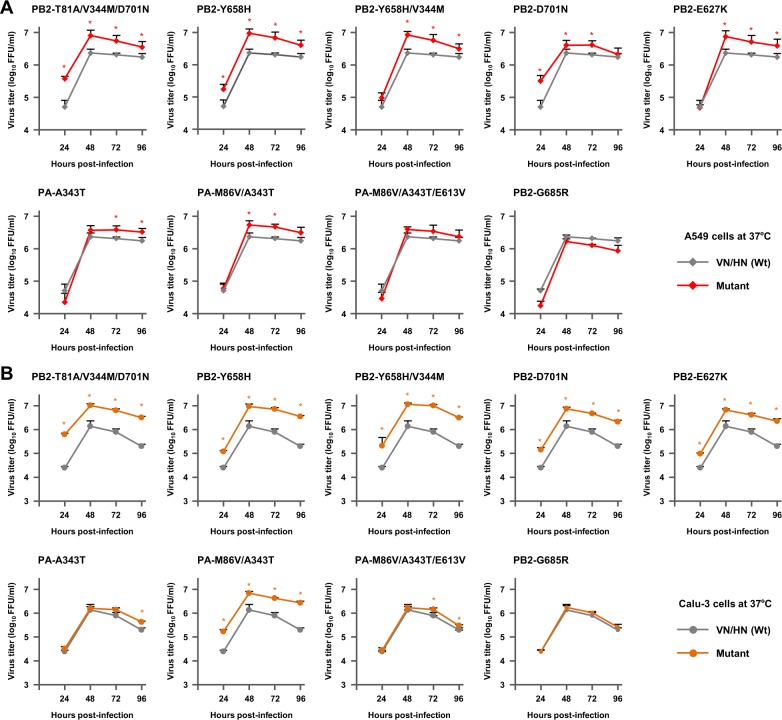
Effect of H5N1 polymerase mutations on viral growth in human airway epithelial cells at 37 °C. (**A**) A549 cells and (**B**) Calu-3 cells were infected with the indicated VN/HN viruses at an MOI of 0.03 and incubated at 37 °C. The culture supernatants were harvested at the indicated times and assayed by focus-forming assays to determine the progeny virus titers. Each data point is the mean ± SD of the log_10_ FFU/ml from three separate experiments. An asterisk indicates a *P* value < 0.01 (ANOVA with Tukey’s multiple comparison test).

**Figure 3:**
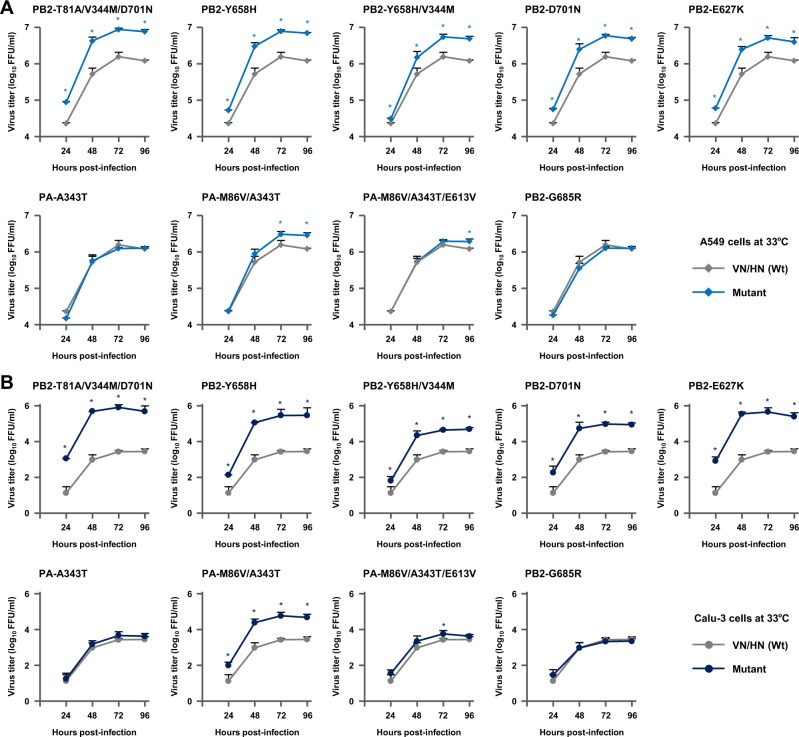
Effect of H5N1 polymerase mutations on viral growth in human airway epithelial cells at 33 °C. (**A**) A549 cells and (**B**) Calu-3 cells were infected with the indicated VN/HN viruses at an MOI of 0.03 and incubated at 33 °C. The culture supernatants were harvested at the indicated times and assayed for focus-forming units to determine the progeny virus titers. Each data point is the mean ± SD of the log_10_ FFU/ml from three separate experiments. An asterisk indicates a *P* value < 0.01 (ANOVA with Tukey’s multiple comparison test).

Human A549 cells and human Calu-3 cells were infected with each of the mutant viruses or with VN/HN (wt) at a multiplicity of infection (MOI) of 0.03, incubated at 33 or 37 °C, and virus titers were determined for 96 h post-infection. In human cells, most of the mutant viruses produced significantly higher progeny virus titers than VN/HN (wt) at all the time points. At 37 °C, the PB2-T81A/V344M/D701N, PB2-Y658H, PB2-Y658H/V344M and PA-M86V/A343T mutants produced progeny virus titers that were up to 4.0-fold higher in A549 cells and 8.3-fold higher in Calu-3 cells, compared to VN/HN (wt) (Fig. 2A and B). Progeny virus production by these mutants at 33 °C was similar to that at 37 °C, but with greater differences between the mutants and wt at 33 °C. The progeny virus titers produced by the mutants were up to 31.2-fold higher in A549 cells and 298.4-fold higher in Calu-3 cells, with the highest increase produced by the PB2-T81A/V344M/D701N mutant (Fig. 3A and B). The PB2-T81A/V344M/D701N and PA-M86V/A343T multiple mutations showed synergistic effects on viral replication compared with the corresponding single mutations both at 37 and 33 °C (Fig. S3A–D, respectively): the maximum progeny virus titers were PB2-D701N < PB2-T81A/V344M/D701N (P < 0.01 by ANOVA with Tukey’s multiple comparison test) and PA-A343T < PA-M86V/A343T (P < 0.01 by ANOVA with Tukey’s multiple comparison test). The effects of these single and multiple mutations on viral replication in human cells were comparable to that produced by the PB2-D701N mutation and, in some conditions, comparable to that produced by the PB2-E627K mutation. In contrast, viral growth of the PB2-G685R putative negative control mutation was indistinguishable from that of VN/HN (wt), both at 33 and 37 °C, although PB2-G685R is located in the PB2-627 domain. These results indicated that the increased polymerase activity of the selected mutations was significant in their adaptation for virus replication in human cells.

**Figure 4:**
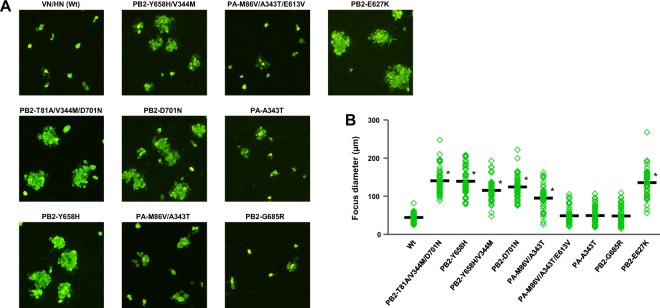
Effect of H5N1 polymerase mutations on foci sizes. (**A**) MDCK cells (90% confluent in 96-well plates) were infected with 100 FFU of the indicated VN/HN viruses. After 48 h incubation at 37 °C, foci sizes were measured by fluorescence microscopy. (**B**) Foci sizes are shown as the average diameter from three separate experiments, with each focus diameter shown as an open diamond. An asterisk indicates a *P* value < 0.01 (ANOVA with Tukey’s multiple comparison test).

**Figure 5:**
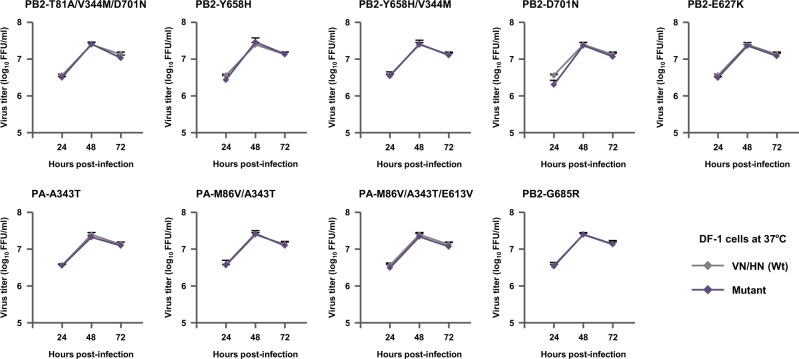
Effect of H5N1 polymerase mutations on viral growth in avian cells. DF-1 cells were infected with the indicated VN/HN viruses at an MOI of 0.01 and incubated at 37 °C. The culture supernatants were harvested at the indicated times and assayed for focus-forming units to determine the progeny virus titers. Each data point is the mean ± SD of the log_10_ FFU/ml from three separate experiments.

To confirm the effects of the selected mutations on H5N1 virus replication, we carried out focus-forming assays in canine MDCK cells and measured the diameters of the foci due to the mutations. Although MDCK cells are not a human cell line, they have been used to investigate human adaptation of AI viruses^16,31,32^. Most mutants produced significantly larger foci diameters than VN/HN (wt) (Fig. 4A and B), and the PB2-T81A/V344M/D701N triple mutation and PA-M86V/A343T double mutation had synergistic effects compared to the corresponding single mutations (Fig. S4, P < 0.05 by ANOVA with Tukey’s multiple comparison test), which were correlated with their increased polymerase activity and replication in human cells. In contrast, in avian DF-1 fibroblasts infected with one of the mutant viruses or with VN/HN (wt) at a MOI of 0.01, there was little difference between the progeny virus titers produced by the mutants and by VN/HN (wt) at all the time points studied (Fig. 5). These results showed that the H5N1 viral mutations selected in patients had a significant effect, alone or in combination with other mutations, in increasing viral replication in human airway cells but not in avian cells, indicating a species-specific role for these mutations in H5N1 virus adaptation to humans.

### Effect of H5N1 polymerase mutations on viral replication in mice *in vivo*

**Figure 6:**
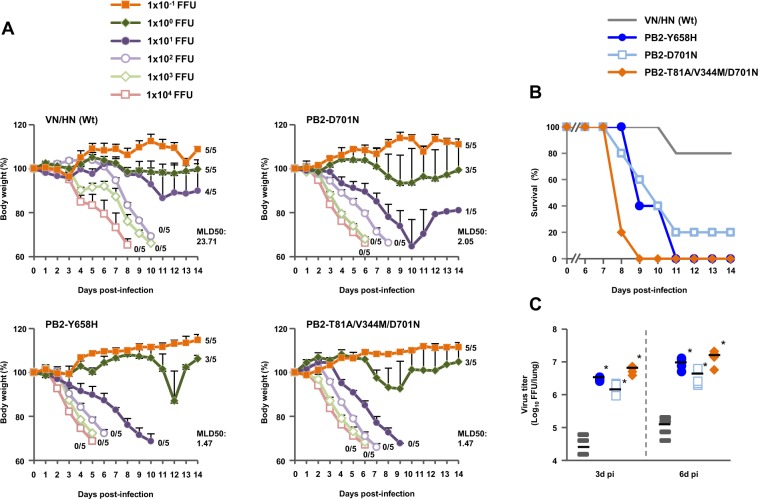
Effect of H5N1 polymerase mutations on mortality and weight loss of infected mice. Five-week-old BALB/c mice were inoculated intranasally with serial 10-fold dilutions of the indicated VN/HN viruses. (**A**) Body weight of mice (5 mice per group) infected with the indicated viruses was monitored for 14 d post-infection. The mean ± SD of the percent body weight change for each group of infected mice is shown. The numbers on the curves show the numbers of surviving animals. (**B**) Survival of mice (5 mice per group) infected with 10 FFU of the indicated viruses. Mortality was calculated including mice that were sacrificed after they had lost more than 30% of their body weight. (**C**) Effect of H5N1 polymerase mutations on virus yields in infected mice lungs. Virus titers in the lungs of five-week-old BALB/c mice (5 mice per group) infected with 100 FFU of the indicated VN/HN viruses were measured at 3 d (left) and 6 d (right) post-infection. Each symbol marks the titer in an individual mouse. An asterisk indicates a *P* value < 0.01 (ANOVA with Tukey’s multiple comparison test).

BALB/c mice were inoculated intranasally with serial dilutions of three mutant viruses that were selected because they produced the most significant increase in both viral polymerase activity and replication in human cells, and monitored daily for weight loss and mortality. No clinical effect was observed in mice infected with 1 focus-forming unit (FFU) VN/HN (wt) during the 14 d observation period (Fig. 6A). In contrast, mice infected with 1 FFU of each of the three mutants showed some weight loss. Most mice infected with 10 FFU VN/HN (wt) survived, but mice infected with 10 FFU of each of the mutants started dying several days after infection. Therefore, survival of the infected mice was correlated with weight loss (Fig. 6B). The mutants were substantially more lethal than VN/HN (wt): the 50% mouse lethal dose (MLD_50_) was 1.47 FFU for the PB2-Y658H mutant, 2.05 FFU for the PB2-D701N mutant and 1.47 FFU for the PB2-T81A/V344M/D701N mutant, which were as much as 16.1-fold less than the MLD_50_ of 23.71 FFU for VN/HN (wt). In agreement with these results, the virus yield in the lungs of mice infected with 100 FFU of the mutants was up to over 100 times greater than with VN/HN (wt) at both 3 and 6 d post-infection (Fig. 6C), with synergistic effects by the PB2-T81A/V344M/D701N triple mutation on viral replication (PB2-D701N < PB2-T81A/V344M/D701N, P < 0.01 by ANOVA with Tukey’s multiple comparison test). These results indicated that mutations that enhanced viral replication in human airway cells *in vitro* contributed to increased viral replication in mice *in vivo* and, of the mutations identified in this study, PB2-Y658H and PB2-T81A/V344M/D701N had the greatest effects on viral virulence in mammals.

### Structural model of the VN/HN polymerase complex

**Figure 7:**
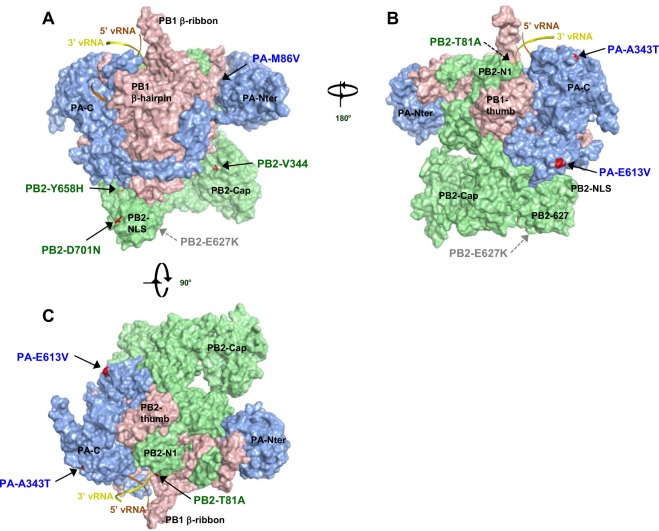
Structural model of the VN/HN polymerase complex. Structural model of the VN/HN heterotrimeric polymerase complex bound to the vRNA promoter. (**A**–**C**) Surface view of the EG/D1 structure is color-coded showing PB2 (green), PB1 (pink), PA (blue) and the mutations in this study (red). Structures in (**A**) and (**B**) differ by 180° in orientation, and structures in (**A**) and (**C**) differ by 90° in orientation.

Models of the VN/HN polymerase complex structure were generated from a crystal structure of the influenza virus polymerase (Protein Data Bank ID code 4WSB)^33^. Our models showed that the mutations that produced increased H5N1 polymerase activity in human cells were located in several domains of the polymerase subunits other than the PB2-627 domain (Fig. 7)^34–37^. Of the PB2 mutations, T81A was in the N1-subdomain and close to the vRNA promoter, V344M was in the Cap-binding domain, Y658H was in the PB2-627 domain and D701N was in the PB2-NLS domain. For PA mutations, PA-M86V was in the PA-endonuclease domain, and A343T and E613V were in the PA-C terminal domain. All the mutations were exposed at the protein surface, except PA-M86V which was at the inter-subunit interface between the PA N-terminal region and the PB C-ext domain.

## Discussion

Previous studies to identify human adaptation mutations in influenza viruses have typically relied on comparison of viruses with high and low pathogenicity in mammals. High-throughput screening now allows analysis of large numbers of mutations and identification of previously unknown human adaptation mutations^17,24,31^. In this study, we targeted a geographically and chronologically related group of polymerase mutants identified in human H5N1 strains isolated in Southern China, Vietnam and Cambodia during 2008–2012. This targeted bioinformatics approach enabled us to identify a number of polymerase gene mutations in H5N1 viruses that had not been previously reported to be human adaptation mutations, in addition to the previously reported PB2-D701N human adaptation mutation. These mutations increased the polymerase activity and replication of clade 2.3.4 viruses in both human cells and mouse lungs to levels similar to the PB2-D701N and PB2-E627K single mutations, which are known human adaptation polymerase mutations. The identification of previously reported and unreported human adaptation mutations in H5N1 viruses confirmed the applicability of our experimental approach and the design of this study. Although the effects of the mutations identified in this study on polymerase activity were relatively low (i.e., less than 5-fold) in our minigenome assay system compared with other studies^14,15,31^, this may have been due to differences in the genetic background used to generate recombinant viruses and to the assay conditions used in these studies.

The adaptation of AI virus polymerase in mammals has been studied extensively and several mutations have been reported to be related to viral cross-species transmission and adaptation to humans. Among these mutations, PB2-E627K and, to lesser extent, PB2-D701N have been described as being critical for avian H5N1 virus replication in mammalian hosts^7,10–12,19,20^. However, in most cases, these mutations were detected as single mutations, implying that they did not have to act synergistically with other mutations. In fact, the PB2-D701N and PB2-Q591R mutations alone, but not combined with the PB2-E627K mutation, produced a replication advantage for the avian H5N1 virus polymerase during human cell infection^13^. In contrast, in this study we found synergistic effects with the PB2-T81A/V344M/D701N and PA-M86V/A343T multiple mutations, producing an additional increase in viral replication in human cells and mouse lungs. The PB2-T81A, PB2-V344M and PB2-T81A/V344M mutations alone had little effect but increased polymerase activity when the two mutations were combined with the PB2-D701N mutation (Figs 1 and S1), implying that both the PB2-T81A and PB2-V344M mutations probably need to cooperate with the PB2-D701N mutation to produce a synergistic effect. Likewise, the PA-M86V mutation alone had little effect on polymerase activity but had a synergistic effect with the PA-A343T mutation. This is in agreement with recent studies showing that several polymerase mutations act cooperatively with PB2-E627K to increase viral growth in mammals^8,17^. These findings suggested that avian H5N1 viruses could acquire multiple human adaptation polymerase mutations in infected patients and that these mutations may act synergistically in optimizing viral fitness to infect humans.

In this study, we identified human adaptation mutations that acted to increase polymerase activity in the genetic background of both clade 2.3.4 and clade 2.2.1 viruses, although clade 2.2.1 viruses are geographically and phylogenetically far from Asian H5N1 clades. This implied that the mutations enabled viruses in different H5N1 genetic lineages to increase viral replication in human cells, although not all the mutations may have the same effects in other clades. However, most of the mutations that significantly increased clade 2.3.4 virus replication in human cells were in the PB2 and PA genes, which was in contrast to our previous study showing that human clinical isolates of clade 2.2.1 viruses had acquired multiple human adaptation polymerase mutations in the PB1 gene^24^. The mechanism(s) underlying the differences in the location of human adaptation polymerase mutations remains unknown. However, the different locations may be due to the different genetic backgrounds of the Asian H5N1 virus clades and the Egyptian clade 2.2.1 viruses. In particular, it should be noted that all Egyptian clade 2.2.1 viruses have the PB2-E627K mutation, whereas all avian viruses in the Asian H5N1 clades, including clade 2.3.4, clade 2.3.2 and clade 1.1 viruses, have the PB2-E627 residue.

The PB2-627 domain has been reported to be a frequent host-adaptation site in the AI polymerase^7,10^, but the exact mode-of-action of the polymerase domains for host adaptation remains unknown. In contrast, our database search approach identified a number of human-adaptation mutations that were scattered over the entire polymerase complex, besides the PB2-627 domain. Although one of the human adaptation mutations identified in this study, PB2-Y658H, was located within the PB2-627 domain, it was at a substantial distance from PB2-627 and did not form a structural site with the PB2-627 residue. In addition, mutations that acted synergistically to increase viral replication were not located near one another in the PB2 structure. This was consistent with previous reports showing that H5N1 viruses could acquire human adaptation polymerase mutations at sites other than in the well-characterized PB2-627 domain^8,17^. The mutations identified in this study were also located on the surface of discrete domains of the polymerase complex or at the inter-subunit interface of the polymerase subunits. In addition, a number of the mutations in PB2 were clustered within the NLS and the Cap-binding domains. This suggested that the mutations may mediate the interaction of the polymerase complex with host factors or among the polymerase subunits. Previous studies reported that PB2-E627K in the 627 domain and PB2-D701N in the NLS domain affected the interaction between the viral polymerase and host importin-α isoforms to adapt mammalian machinery^19,20^. Also, some mutant residues may not make direct protein contacts but instead may affect protein flexibility, to help other protein regions maintain polymerase activity or to promote interactions with other protein domains or host factors. These considerations implied that the molecular basis underlying human adaptation by H5N1 polymerase mutations may be multifactorial. However, our structural models may be incomplete, because the viral polymerase complex has been found to be flexible, which allows it to adopt different conformations. Therefore, the exact mechanism(s) by which mutations affect human adaptation of AI viruses needs to be further investigated.

The human adaptation mutations identified here have been detected sporadically in H5N1 viruses. The PB2-T81A/V344M/D701N triple mutation has only been detected in influenza virus A/Hunan/1/2008 (clade 2.3.4)^38^. The PB2-Y658H mutation has been reported in three human clade 2.3.4.1 virus strains (A/Guizhou/1/2009, A/Hunan/1/2009 and A/Hunan/2/2009) and in four avian clade 2.3.4.1 virus strains (A/chicken/Lao/LH1/2010, A/duck/Lao/19/2010, A/duck/Lao/463/2010 and A/duck/Lao/567/2010)^39^. The PB2-Y658H mutation has also been detected in one avian H9N2 virus strain (A/chicken/Jiangsu/DT0112/2012)^40^, one avian H5N8 virus strain (A/quail/California/K1400794/2014)^41^ and seven avian H5N6 virus strains (A/mute swan/Kyoto/1T/2016, A/mute swan/Kyoto/2T/2016, A/mute swan/Kyoto/3T/2016, A/mute swan/Kyoto/4T/2016, A/mute swan/Kyoto/5T/2016, A/mute swan/Kyoto/6T/2016 and A/mute swan/Kyoto/8T/2016)^42^. Neither the PB2-T81A/V344M/D701N nor the PB2-Y658H mutation has been found in recent (i.e., during 2001–2017) isolates of human seasonal H1N1 and H3N2 viruses. However, recent studies have suggested the possible relevance of these mutations to the human adaptation of AI viruses^13,14,43^. In a high-throughput screening study of random mutagenesis libraries, the PB2-Y658S amino acid change was identified as a mutation that increased H5N1 replication in human cells^14^. PB2 residue 658 also forms a host-specific structural pocket along with residues 613 and 661 that may be involved in avian to human adaptation^43^. PB2-V344M has been reported to be one of the mutations in influenza virus A/Indonesia/UT3006/2005 (clade 2.1.3) that increased viral replication in human cells^13^. However, in these previous studies, the effect of these mutations on host adaptation was not assessed because of their relatively low prevalence in H5N1 viruses.

This study was limited by the relatively small number of sequences of clinical human clade 1.1, clade 2.3.2.1 and clade 2.3.4 strains isolated in 2008–2012 in databases, although all of the available sequences of human strains isolated during 2008–2012 were analyzed in this study. Furthermore, host adaptation of influenza viruses involves polygenetic traits, implying that AI viruses probably require adaptation mutations in addition to those in PB2 (e.g., in other genes such as the HA gene^4,44^ and the NS gene^45,46^) to become pandemic. Future database searches should be done using larger sequence data sets, since larger numbers of AI sequences from human isolates have recently been deposited in multiple public databases and should be available for bioinformatics analyses.

In conclusion, we have identified a number of putative human adaptation polymerase mutations that were selected in infected patients using a database search targeting geographically and chronologically close sequences of H5N1 viruses from Southern China, Vietnam and Cambodia. Since all the mutations identified here have been found in natural isolates, these human adaptation mutations may (re) emerge in novel strains and perhaps facilitate virus adaptation to mammals. In fact, novel H5N6 viruses carrying clade 2.3.4 internal genes have emerged since 2013 and caused outbreaks in East Asia and Southeast Asia^42,47^. Some viruses isolated from these outbreaks had the PB2-Y658H polymerase human adaptation mutation that was identified in this study. The results presented here should help in influenza virus surveillance efforts and contribute to understanding the mechanism of AI virus adaptation to new hosts.

## Materials and Methods

### Ethics statement

All animal experiments were conducted in compliance with Japanese legislation (Act on Welfare and Management of Animals, 1973, revised in 2012) and guidelines under the jurisdiction of the Ministry of Education, Culture, Sports, Science & Technology in Japan, and were approved by the Animal Experiment Committee of the Kyoto Prefectural University of Medicine (Approval number M29-554). Mice that lost more than 30% of their original weight were humanely euthanized.

### Biosecurity and biosafety

All experiments with live H5N1 viruses were performed at enhanced Biosafety Level 3+ (BSL3+) in the Research Institute for Microbial Diseases, Osaka University and Kyoto Prefectural University. All studies with recombinant DNAs were approved by the Biological Safety Committee of Osaka University (approval number 3439) and Kyoto Prefectural University of Medicine (approval number 25–2) after risk assessments were conducted by the Living Modified Organisms Committee of Osaka University, Kyoto Prefectural University of Medicine and, when required, by the appropriate minister of Japan.

The BSL3+ facility of the Research Institute for Microbial Diseases, Osaka University, and Kyoto Prefectural University of Medicine consists of negative-pressure laboratories in which all experimental work is carried out in biosafety cabinets. Air exhausted from the class 3 units is filtered by HEPA filters and then leaves the facility via a second set of HEPA filters. The BSL3+ has a dedicated electrical generator in the event of power loss.

Only authorized personnel that have received appropriate training can access the BSL3+ facility. All personnel working in the BSL3+ facility wear a disposable protective, FFP3 facemasks and multiple pairs of gloves. Furthermore, all personnel conducting this study were vaccinated against seasonal and H5N1 influenza viruses. Antiviral drugs are directly available to further mitigate risks upon incidents.

In this study, all the mutations introduced into the recombinant VN/HN virus (clade 2.3.4) have been detected as single or multiple mutations in H5N1 viruses isolated from patients, except the PB2-E627K mutation. Also, the mouse infection study was performed after no increase was observed in the progeny virus titers of the selected viruses carrying the mutations in this study compared to previously published studies. The PB2-E627K mutant virus was excluded from the mouse infection experiments in this study.

### Database search

A database search was conducted as described previously^17,24^. Briefly, sequences of PB2, PB1, PA and NP genes from 222 influenza A virus subtype H5N1 strains isolated in Southern China, Vietnam and Cambodia from 2008–2012 were obtained from the National Center for Biotechnology Information (NCBI) Influenza Virus Resource (www.ncbi.nlm.nih.gov/genomes/FLU/). Duplicate sequences from the same strain and sequences with questionable amino acid translation were removed. Nucleotide sequences of genes with more than 100 amino acids missing at either end were also excluded. These sequences were aligned using the MAFFT program^48^. Polymerase mutations in 24 human and 198 avian H5N1 virus strains were identified by comparing these sequences to a consensus sequence of each protein that was determined from the aligned sequences of all the H5N1 strains downloaded. The prevalence of the mutations in the human and avian virus strains was then calculated and compared between viruses isolated from human and avian hosts. To increase the likelihood of identification of relevant human-adaptation mutations, polymerase mutations that were either in human viruses or were more prevalent in human viruses than in avian viruses (arbitrary cutoff value of over 4 fold) were included.

### Cells

Human embryonic kidney 293T cells, human lung carcinoma A549 cells, canine kidney MDCK cells and quail fibroblast QT-6 cells were maintained in Dulbecco’s Modified Eagle’s Medium or Ham’s F-12K medium supplemented with 10% fetal calf serum at 37 °C in 5% CO_2_. Human bronchial epithelial Calu-3 cells and chicken fibroblast DF-1 cells were maintained in Dulbecco’s Modified Eagle’s Medium supplemented with 10% fetal calf serum at 37 °C in 5% CO_2_. Chicken embryo fibroblasts (CEFs) were prepared from 10-day-old embryonated eggs as described previously^49^.

### Virus preparation

Influenza viruses were grown in 10-day-old embryonated chicken eggs that had been purchased from Shimizu Laboratory Supplies, Japan. The allantoic fluids and culture supernatants were then harvested and stored as seed viruses at −80 °C. Virus purification to produce working stocks was described previously^30,50^. Virus titers were assayed as FFU by focus-forming assays using MDCK cells as described below.

### Focus-forming assays

MDCK cells (90% confluent in 96-well plates) were infected and, after 1 h at 37 °C, the virus inoculum was removed and the cells were washed with PBS and overlaid with 1% methylcellulose in Modified Eagle’s Medium. At 16 h post-infection at 37 °C, the cells were fixed with 4% paraformaldehyde in PBS, incubated with anti-H5N1 virus polyclonal antibody for 1 h at 37 °C, washed 3 times with PBS, and reacted with Alexa Fluor 488 anti-rabbit antibody for 1 h at 37 °C. After 3 washes with PBS, the plates were coated with a 50% glycerol solution. Digital images were taken using an inverted fluorescence microscope ECLIPSE Ti2 system (Nikon) and foci sizes were measured using NIS-Elements software (Nikon).

### Minigenome assays

Minigenome assays based on a dual-luciferase system were performed as described previously^17,50^. Briefly, 293T and QT-6 cells were transfected with plasmids each expressing the PB2, PB1, PA or NP gene of A/Vietnam/HN31432/2008 (VN/HN) or A/duck/Egypt/D1Br/2007 (EG/D1), and a human or chicken polymerase I-driven plasmid expressing firefly luciferase from a virus-like RNA. Cells were also transfected with a plasmid expressing *Renilla* luciferase to monitor transfection efficiencies. Cells were transfected with these plasmids using *Trans*IT-LT1 (Mirus) and incubated at 33 or 37 °C. Firefly luciferase activity values at 48 h post-transfection were normalized relative to the *Renilla* luciferase activity.

### Reverse genetics

Recombinant viruses were generated with a plasmid-based reverse genetics system in the VN/HN (wt) virus genetic background as described previously^51,52^. All viruses were sequenced to ensure the absence of unwanted mutations.

### Viral growth kinetics in cultured cells

A549 and Calu-3 cells were infected in triplicate with viruses at an MOI of 0.03, and DF-1 cells were infected in triplicate with viruses at an MOI of 0.01. After 1 h at 37 °C, the cells were washed with PBS, followed by further incubation at 33 or 37 °C. At the indicated times post-infection, virus titers in the cell culture supernatants were assayed by focus-forming assays.

### Experimental infections in mice

Five-week-old female BALB/C mice (Japan SLC), under mixed anesthesia (medetomidine-butorphanol-midazolam), were intranasally inoculated with 10^−1^ to 10^4^ FFU of viruses in PBS. The mice body weight and survival were monitored daily for 14 d. Mice that lost more than 30% of their original weight were euthanized. In addition, at 3 and 6 d after inoculation with 1 × 10^2^ FFU virus, mouse lungs were collected and virus titers were assayed as FFU in MDCK cells.

### Homology modeling of VN/HN polymerase mutants

Homology modeling of VN/HN polymerase used the crystal structure of the heterotrimeric polymerase complex of influenza virus A/little yellow-shouldered bat/Guatemala/060/2010 (H17N10) (Protein Data Bank ID code 4WSB)^33^ as a template, as described previously^17^.

### Statistical analysis

Statistical analysis was carried out using GraphPad Prism Version 6 software (GraphPad Software Inc.).

## Electronic supplementary material

Supplementary Information
