# Supplementary material for: Multiple polymerase gene mutations for human adaptation occurring in Asian H5N1 influenza virus clinical isolates
Source: Sci Rep. 2018 Aug 30;8:13066. doi: 10.1038/s41598-018-31397-3 (PMC6117316; doi:10.1038/s41598-018-31397-3)
Supplement: Supplementary file 1 — Supplementary Information [file 41598_2018_31397_MOESM1_ESM.pdf]

Supplementary Information for

Multiple polymerase gene mutations for human adaptation occurring in Asian H5N1 influenza virus clinical isolates

Yasuha Arai[a,b], Norihito Kawashita[c,d], Kozue Hotta[e,f], Phuong Vu Mai Hoang[g], Hang Le Khanh Nguyen[g], Thach Co Nguyen[g], Cuong Duc Vuong[g], Thanh Thi Le[g], Mai Thi Quynh Le[g], Kosuke Soda[h], Madiha S. Ibrahim[i], Tomo Daidoji[a], Tatsuya Takagi[d], Tatsuo Shioda[b], Takaaki Nakaya[a], Toshihiro Ito[h], Futoshi Hasebe[e], Yohei Watanabe[a,b]

[a]Department of Infectious Diseases, Graduate School of Medicine, Kyoto Prefectural University of Medicine, Kyoto, Japan, [b]Department of Viral Infections, Research Institute for Microbial Diseases, Osaka University, Osaka, Japan, [c]Graduate School of Science and Engineering, Kindai University, Osaka, Japan, [d]Graduate School of Pharmaceutical Science, Osaka University, Osaka, Japan, [e]Vietnam Research Station, Center for Infectious Disease Research in Asia and Africa, Institute of Tropical Medicine, Nagasaki University, Hanoi, Vietnam, [f]Laboratory of Veterinary Public Health, Department of Veterinary Medical Sciences, Graduate School of Agricultural and Life Science, The University of Tokyo, Tokyo, Japan, [g]Department of Virology, National Institute of Hygiene and Epidemiology, Hanoi, Vietnam, [h]Avian Zoonosis Research Center, Faculty of Agriculture, Tottori University, Tottori, Japan, [i]Department of Microbiology and Immunology, Faculty of Veterinary Medicine, Damanhour University, Damanhour, Egypt

Address corresponding to Yohei Watanabe, nabe@koto.kpu-m.ac.jp

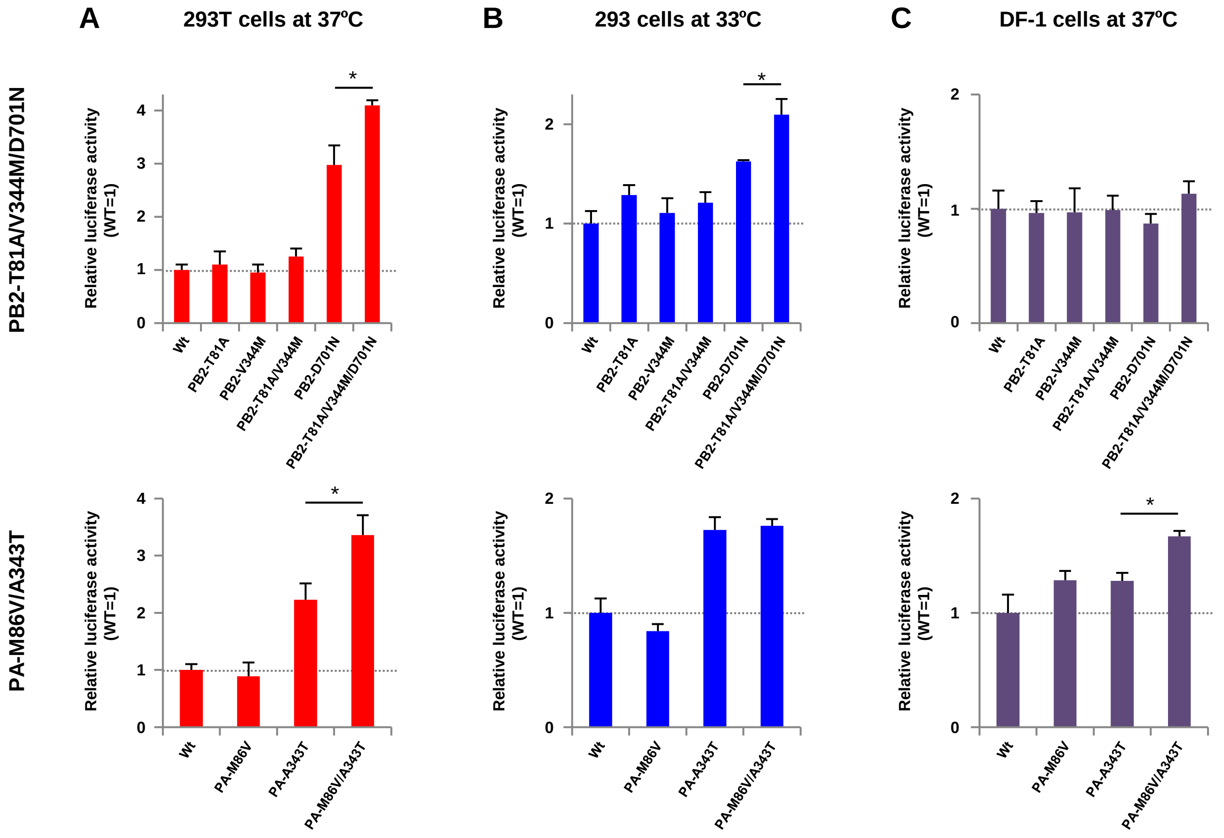

Fig. S1. Synergistic effect of multiple mutations on polymerase activity. To clarify the significance of the PB2-T81A/V344M/D701N and PA-M86V/A343T multiple mutations on polymerase activity, the polymerase activity data of the indicated VN/HN mutants (Fig. 1) were compared. (A) 293T cells at 37ºC. (B) 293T cells at 33ºC. (C) QT-6 cells at 37ºC. An asterisk indicates a *P* value < 0.01 (ANOVA with Tukey's multiple comparison test).

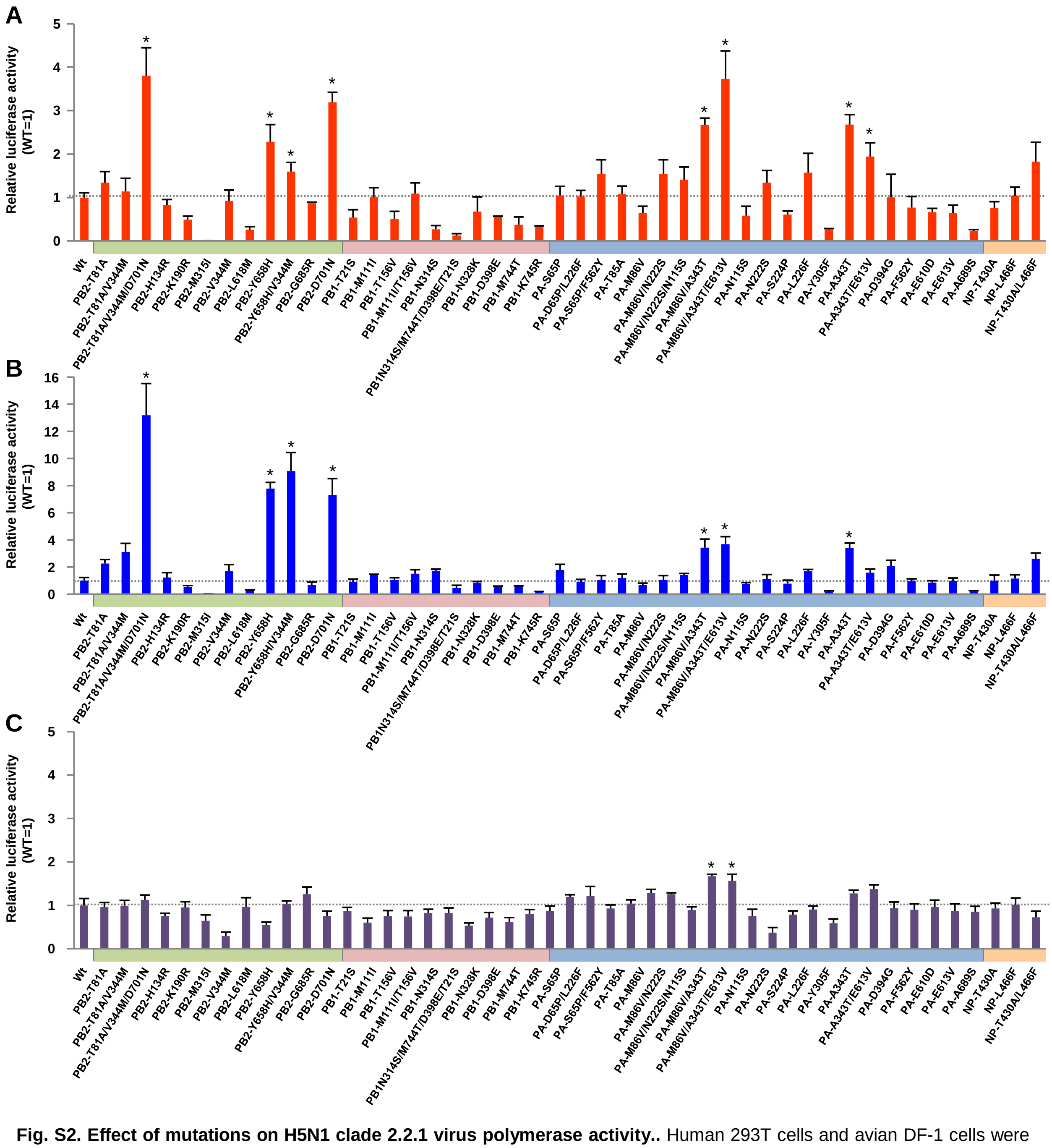

Fig. S2. Effect of mutations on H5N1 clade 2.2.1 virus polymerase activity.. Human 293T cells and avian DF-1 cells were transfected with plasmids expressing EG/D1 PB2, PB1, PA or NP with the indicated single or multiple mutations, a human or chicken polymerase I-driven plasmid expressing a vRNA-oriented luciferase reporter gene, and a plasmid expressing *Renilla* luciferase as an internal control. After 48 h incubation at 33 or 37ºC, luciferase activities were measured, normalized to the internal *Renilla* luciferase activity, and expressed relative to the results for VN/HN (wt). (A) EG/D1 polymerase activity at 37ºC in 293T cells. (B) EG/D1 polymerase activity at 33ºC in 293T cells. (C) EG/D1 polymerase activity at 37 ºC in DF-1 cells. Colors on each x-axis indicate the different virus genes. An asterisk indicates a *P* value < 0.01 (ANOVA with Tukey's multiple comparison test). Asterisks for mutations with negative effects on polymerase activity were omitted for clarity.

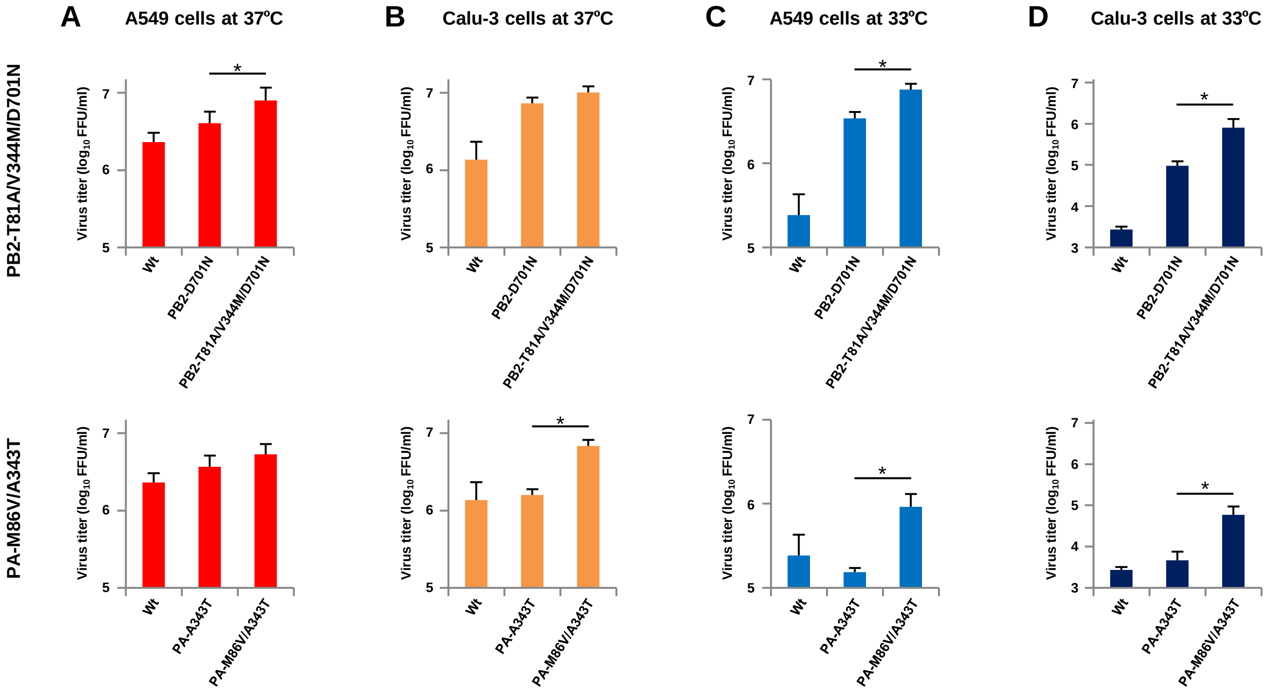

Fig. S3. Synergistic effect of multiple mutations on viral replication in human airway epithelial cells. To clarify the significance of the PB2-T81A/V344M/D701N and PA-M86V/A343T multiple mutations in viral replication, virus titer data at 48 or 72 h post-infection for the indicated VN/HN mutants (Fig. 2) were compared. (A) A549 cells at 37ºC. (B) Calu-3 cells at 37ºC. (C) A549 cells at 33ºC. (D) Calu-3 cells at 33ºC. An asterisk indicates a P value < 0.01 (ANOVA with Tukey's multiple comparison test).

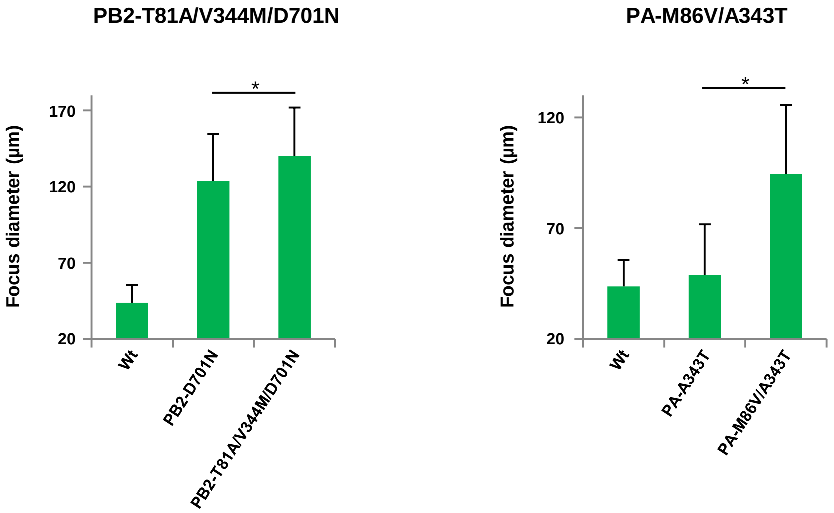

Fig. S4. Synergistic effect of multiple mutations on foci sizes. To clarify the significance of the PB2-T81A/V344M/D701N and PA-M86V/A343T multiple mutations on foci sizes, foci size data for the indicated VN/HN mutants (Fig. 4) were compared. An asterisk indicates a *P* value < 0.01 (ANOVA with Tukey's multiple comparison test).

Table S1. Mutations in the polymerase sequence of 24 H5N1 viruses isolated from patients in Asia and identified by an NCBI database search.

Clade	Virus Strain	Subtype	Passage history†	Collection date	Mutation in gene			
					PB2	PB1	PA	NP
2.3.2.1	A/Guangxi/1/2009	H5N1	E1	2009-01-19		M111I/N328K	L226F	
2.3.2.1	A/Hong Kong/6841/2010	H5N1	-	2010-11-16	G685R		N115S	
2.3.2.1	A/Hubei/1/2010	H5N1	E1	2010-05-21				
2.3.4	A/Guangdong/1/2008	H5N1	E2	2008-02-16	T81A/V344M			
2.3.4	A/Guangxi/1/2008	H5N1	E2	2008-02-12	M315I/L618M	M111I/T156V/K745R	L226F	
2.3.4	A/Hunan/1/2008	H5N1	E2	2008-01-16	T81A/K190R/V344M/D701N		A343T/E613V/A689S	
2.3.4	A/Shandong/1/2009	H5N1	E1	2009-01-05	T81A/V344M	N328K	D394G	
2.3.4	A/Xinjiang/1/2009	H5N1	E2	2009-01-10			N222S/F562Y	
2.3.4.1	A/Guizhou/1/2009	H5N1	E2	2009-01-15	Y658H	T21S/N314S/D398E/M744T	N115S/N222S	T430A/L466F
2.3.4.1	A/Hunan/1/2009	H5N1	E3	2009-01-08	K190R/Y658H		T85A/Y305F	
2.3.4.1	A/Hunan/2/2009	H5N1	E2	2009-01-23	V344M/Y658H	T21S/N314S/D398E/M744T		T430A/L466F
2.3.4	A/Beijing/1/2009	H5N1	E3	2008-12-04			T85A/Y305F	
2.3.4.3	A/Vietnam/HN31432M/2008	H5N1	C2	2008-02-21			D394G/F562Y	
2.3.4.3	A/Vietnam/UT31394II/2008	H5N1	C2	2008-01-17	M315I/L618M	M111I/T156V/K745R		
2.3.4.3	A/Vietnam/UT31412II/2008	H5N1	C2	2008-02-09	L618M	K745R	A343T/E613V/A689S	T430A
2.3.4.3	A/Vietnam/UT31413II/2008	H5N1	C2	2008-02-13	L618M	K745R	A343T/E613V	
1.1	A/Cambodia/S1211394/2008	H5N1	-	2008-12-11	G685R		A343T/E613V	
1.1	A/Cambodia/U0417030/2010	H5N1	-	2010-04-18			D394G	
1.1	A/Cambodia/V0203306/2011	H5N1	-	2011-02-03				
1.1	A/Cambodia/V0219301/2011	H5N1	-	2011-02-17				
1.1	A/Cambodia/V0401301/2011	H5N1	-	2011-03-31			S224P	
1.1	A/Cambodia/V0417301/2011	H5N1	-	2011-04-16				
1.1	A/Cambodia/V0606311/2011	H5N1	-	2011-06-06			S224P	
1.1	A/Cambodia/W0112303/2012	H5N1	-	2012-01-10	D701N			

† C, cultured cells; E, eggs. The number after each letter indicates the number of passages of the strain.

- No information
